# Probiotics and Prebiotics for the Treatment of Irritable Bowel Syndrome—A Narrative Review

**DOI:** 10.3390/jcm13216337

**Published:** 2024-10-23

**Authors:** Carolina Marques Lopes, Cristina Sofia de Jesus Monteiro, Ana Paula Duarte, Jorge Luiz dos Santos

**Affiliations:** 1 Faculdade de Ciências da Saúde, Universidade da Beira Interior, 6200-506 Covilhã, Portugal; carolina.marques.lopes@ubi.pt (C.M.L.); csjmonteiro79@gmail.com (C.S.d.J.M.); apcd@ubi.pt (A.P.D.); 2 CICS-UBI-Health Sciences Research Centre, Faculdade de Ciências da Saúde, Universidade da Beira Interior, 6200-506 Covilhã, Portugal; 3 Academic Clinical Center of Beiras, Faculdade de Ciências da Saúde, Universidade da Beira Interior, 6200-506 Covilhã, Portugal; 4 UFBI—Pharmacovigilance Unit of Beira Interior, Faculdade de Ciências da Saúde, Universidade da Beira Interior, 6200-506 Covilhã, Portugal

**Keywords:** prebiotics, probiotics, functional gastrointestinal disorders, irritable bowel syndrome, efficacy

## Abstract

**Background/Objectives:** Gastrointestinal functional disorders (GFDs), including irritable bowel syndrome (IBS), are imbalances in the gut–brain axis characterized by persistence of symptoms in the abdominal area. Probiotics are live microorganisms that provide benefits to the health of their hosts when administered in adequate amounts, while prebiotics are a substrate that is selectively used by host microorganisms. This narrative review aimed to evaluate the effectiveness of prebiotics and probiotics mostly in irritable bowel syndrome, particularly on issues such as the interaction between these products and the gut microbiota, the duration of supplementation and long-term effects, the definition of ideal dosages, and the regulation and quality control of these products. **Methods:** A bibliographic search was carried out in indexed databases and articles published within 10 years before the beginning of the study and publications in English language, which investigated the specific theme of the study were considered. Papers dealing with topics not covered by the research questions, or presenting errors related with the wrong population or the wrong methods, as well as experimental studies and case reviews were excluded. Fifty-five articles were selected, initially in isolation by the authors and, afterward, under consensus. **Results:** It was possible to observe the effectiveness mainly of probiotics, in improving specific symptoms of the respective disorder; however, the available data remain unclear due to limitations concerning samples and methods of the studies evaluated. **Conclusions:** Despite evidence suggestive of therapeutic efficacy, additional multicenter randomized controlled trials (RCTs) with better defined protocols are still necessary to fill in the gaps in this subject, define measures to ensure the safe administration of these products, and confirm their therapeutic potential.

## 1. Introduction

The attention regarding the worldwide interest in the therapeutic use of probiotics in medicine started in Japan in 1935, when Dr. Minoru Shirota isolated a strain of *Lacticaseibacillus paracasei* to deal with outbreaks of diarrhea [1,2].

Currently, probiotics are defined as “live microorganisms that, when administered in adequate amounts, confer a health benefit to the host” and prebiotics as “a selectively fermented ingredient that allow specific changes in the composition and/or activity of the gastrointestinal microbiota thus conferring benefits to the health of the host”. Synbiotics, on the other hand, are defined as “a mixture of live microorganisms that confer a benefit to the host’s health” and are considered associations of probiotics and prebiotics that can act complementarily or synergistically [1,2,3,4]. Within the group of prebiotics, non-starch polysaccharides, lactulose, and breast milk oligosaccharides stand out. The probiotic species that seem to present some benefits in the studies developed are *Bifidobacterium* (*adolescentis*, *animalis*, *bifidum*, *breve* and *longum*) and *Lactobacillus* (*acidophilus*, *casei*, *fermentum*, *gasseri*, *johnsonii*, *paracasei*, *plantarum*, *rhamnosus* and *salivarius*) [1,3,4].

Studies have emerged, although with limited results, suggesting that probiotics are useful in supporting a healthy digestive tract, acting in clinical situations such as: persistent diarrhea in children [5], diarrhea associated with *Clostridium difficile* [6], infectious diarrhea [7], necrotizing enterocolitis [8], the eradication of *Helicobacter pylori* [9], in functional abdominal pain disorders in children [10], functional constipation [11] and IBS [12]. As far as prebiotics are concerned, they seem to have benefits in situations such as: metabolic health [13], intestinal health in babies [14], IBS [15], inflammatory bowel disease [16], and immune function [17].

Despite the proposed benefits, the mode of action of these products is not yet well defined. The suggested mechanisms for their efficacy include downregulation of inflammation at the epithelial level through inhibition of the effects of phosphorylation over the inflammatory process [18], protection of the mucosal barrier integrity [19], elimination of pathogenic microorganisms, by competing for nutritional resources such as iron [20], or by blocking the adhesion of pathogens to binding sites [21]. An additional putative mechanism is protection against toxins by their inactivation [22]. On the other hand, prebiotics seem able to enhance activity of beneficial bacteria through induction of molecules with immunomodulatory properties, thus influencing the inactivation of pro-inflammatory cytokines, and resulting in decreased harmful metabolic activities [3,23].

In addition to doubts concerning the mechanisms of action, the main adverse effects and contraindications of probiotics and prebiotics are also not fully defined. Table 1 describes some adverse effects reported due to the administration of probiotics in certain groups of subjects.

Given the existence of the adverse effects described in Table 1, it has been recommended that caution should be exercised in the use of the products in question in immunocompromised people, pregnant women, premature infants, and in some conditions such short bowel syndrome, use of central venous catheter, or heart valve. Larger multicenter, prospective, and randomized studies are needed concerning this subject [40,41].

Examples of other issues that remain incompletely understood are as follows: the fact that the long-term effects of using these products are not known; the role of microbiota in human health is not well understood; the prediction of interactions between probiotic strains and gut microbiota is unavailable; and it is also necessary to determine the adequate duration of supplementation, as well as defining the adequate dosages. The fact that regulatory and quality control issues are not yet ideal hampers the correct interpretation of clinical data and increases the need for enlightening communication between consumers and healthcare professionals about the role and definition of prebiotic and probiotic products [1,3,4,42,43].

**Figure 1:**
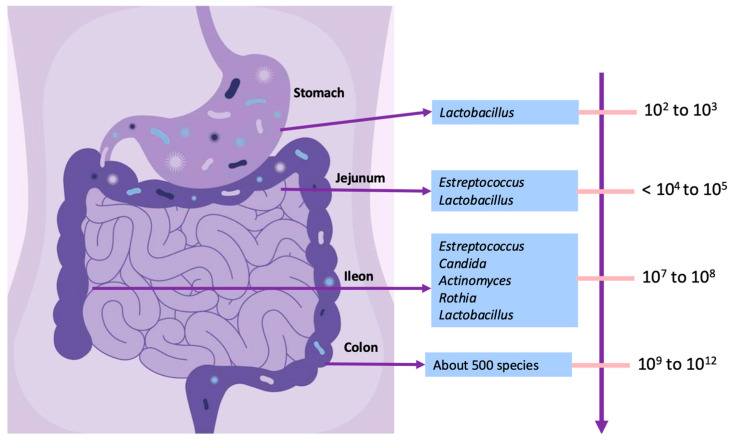
Variation in microbial content along the gastrointestinal tract. Adapted from Villmones et al., Shintani et al., Kastl et al. [48,49,50].

Understanding the role of prebiotics and probiotics in gastrointestinal disorders requires knowledge of the gut microbiota, which represents the population of microorganisms that colonize a given site and establish the microbiome, including bacteria, fungi, viruses, protozoa, and archaea [44,45]. Along the length of the gastrointestinal tract, microbial contents vary from a low diversity of a smaller number of microbial species in the stomach to a high diversity of a higher number of microbial cells in the gut, Figure 1 [46,47].

A healthy gut microbiota is predominantly made up of bacteria from two phyla: *Firmicutes* and *Bacteroidetes*, the former being divided into two classes, mostly comprising Gram-positive bacteria, *Bacilli* and *Clostridia*, including genera such as *Clostridium*, *Enterococcus*, *Lactobacillus,* and *Ruminococcus*. Bacteroidetes are mostly Gram-negative bacteria which include the genera *Bacteroides* and *Prevotella*, whereas the phyla of the remaining bacteria are mostly *Proteobacteria*, *Tenericutes*, *Verrucomicrobia*, *Actinobacteria*, *Fusobacteria*, and *Cyanobacteria* [45,46,47]. However, several factors can influence the gut microbiota, culminating in an unhealthy pattern, such as suppression of components of the normal microbiota by the use of broad-spectrum antibiotics. This can culminate in the action of potentially pathogenic microorganisms [51]; or disturbance of the immunological interaction between microbiota and the host, leading to incorrect identification of the normal microbiota as a dysbiotic flora, thus triggering inflammation, and resulting in damage to the intestinal epithelium [52].

Changes in gut microbiota are thought to correlate with several gastrointestinal disorders, including IBS. Some studies, although with conflicting results, have investigated the differences in the composition of gut microbiota from IBS and healthy patients, finding significant dissimilarities between the samples [53].

Gut microbiota presents a symbiotic relationship with its host, a fundamental factor for maintaining body homeostasis. Additionally, there is a bidirectional interaction between the gut with its microbiota and other organs, including the host´s nervous system, the so-called “gut–brain axis”. Situations of disruption from the normal composition of gut microbiota, named dysbiosis, can lead to impairment of the gut–organ axis, resulting in organic disorders [54]. GFDs represent imbalances specifically of the gut–brain axis characterized by the persistence of symptoms without structural or biochemical abnormalities as detected by routine diagnostic tests [55].

Among GFDs, IBS stands out. This is essentially characterized by persistent or recurrent symptoms of abdominal pain, altered bowel function, complaints of flatulence, bloating, nausea, constipation or diarrhea, and anxiety or depression. Other findings may include mucus in the stool and the presence, or sensation of, abdominal distension. Diagnosis of IBS is predominantly based on symptoms and their duration [56,57,58,59].

**Figure 2:**
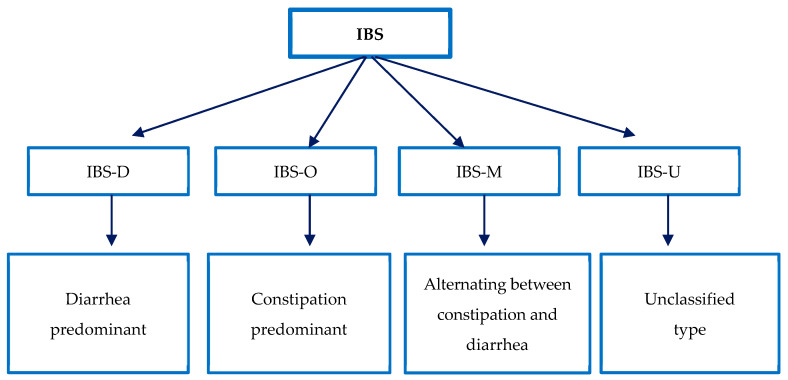
Classification of IBS subtypes. Adapted from “Rome Foundation-Criteria” [59].

The Rome IV criteria classify IBS into four subtypes (Figure 2) considering the predominant intestinal alteration evaluated by the “Bristol Stool Form Scale”. This scale is a tool used to evaluate bowel habits and should be used to record stool consistency, ranging from watery stools to obstipation [59].

IBS is thought to result from the interaction of several factors and is considered a biopsychosocial disease. The mechanisms underlying IBS are not yet fully understood; however, there are dissimilar hypotheses involving altered gastrointestinal motility; increased visceral sensitivity to physiological stimuli, presence of intestinal inflammation, increased intestinal permeability, relationship with previous episodes of gastroenteritis, existence of alterations in the intestinal microflora, bacterial overgrowth, genetics, and the existence of psychosocial dysfunction [59,60,61,62,63,64,65].

Other studies also argue that gluten sensitivity may contribute to food sensitivity in IBS patients and that eliminating the intake of gluten-containing food may lead to an improvement in symptoms [66,67], but the studies present disputable results. On the other hand, there are studies, that argue that it is more beneficial to bet on a diet low in oligosaccharides, disaccharides, monosaccharides and non-fermentable polyols (FODMAPs), described as poorly absorbable carbohydrates that exert an osmotic load in the intestine and are quickly fermented by colon bacteria, resulting in the production of gas, and thus causing abdominal distension, bloating, and pain [68].

Regarding the treatment of IBS, presently, it is mostly based on symptom relief. Within non-pharmacological measures, the low-FODMAP diet seems to stand out as the dietary intervention with the best evidence in the management of IBS, as well as betting on a traditional diet that includes regular and light meals, adequate hydration and reduction of the intake of fats, insoluble fibers, alcohol, caffeine, and foods that promote the formation of gas. Another measure that can be adopted is the practice of regular physical exercise either to improve IBS symptoms or the general state of health [69].

Concerning pharmacological measures, those contained in guidelines and algorithms described in Table 2, aim to develop a therapeutic plan for people with IBS in order to reduce the associated symptoms and consequently improve quality of life.

When pharmacological measures are not effective, studies have shown that psychological and behavioral interventions can contribute to improving the symptoms associated with IBS, with cognitive behavioral therapy being the most studied intervention and the one that seems to be most effective [73,74].

Currently, and as an innovative therapy, some probiotics have emerged as a pharmacological option for IBS, and can be used for diarrhea, constipation, and abdominal pain. There is some evidence that these may be beneficial in controlling the overall symptoms of IBS, with some products containing *Lactobacillus* having been shown to have a positive effect [75,76,77].

This study aimed to study in depth unsolved questions concerning this topic, by updating the available information on the effectiveness of probiotics and prebiotics in GFDs, namely IBS.

## 2. Materials and Methods

In order to evaluate the effectiveness and safety of the use of probiotics and/or prebiotics in GFDs, particularly in the treatment of IBS, a narrative review of the literature was carried out. The bibliographic research was performed in indexed databases, namely, PubMed, Scopus, and Web of Science. The keywords used were “Probiotics” and “Prebiotics”, crossed with the terms “Irritable Bowel Syndrome”, “Functional Gastrointestinal Disorders”, “efficacy”, “effectiveness”, “gut microbiota”, “dose”, and “quality control”. The abstracts concerning the subject under study, after being obtained in indexed medical databases, were included into Rayyan (Intelligent Systematic Review—https://new.rayyan.ai/) and evaluated by the authors, first by each author alone and, afterward, under consensus. Inclusion criteria comprised articles published within 10 years before the beginning of the study; publications in English language, which investigated the specific theme of the study. Exclusion criteria, in addition to not adapting to the previously described inclusion standards, comprised duplicated articles, papers dealing with topics not covered by the research questions, or presenting errors related with the wrong population or the wrong methods, as well as experimental studies and case reviews.

In addition to the aforementioned criteria, other basic and historical bibliographical sources necessary for the adequate understanding on the subject were recommended by the advisor of the present study, irrespective of the year of publication.

## 3. Results and Discussion

Initially, 238 articles were identified, from which 127 articles were selected after the first analysis of all titles and abstracts. After exclusion, the total number of scientific papers included for the preparation of the review was 55 articles, 49 of which RCTs, 4 to systematic reviews and 2 to non-randomized trials.

### 3.1. The Interaction Between Probiotic Strains and the Gut Microbiota Is Related to the Health of Individuals

Of the 49 RCTs used for analysis, fifteen groups were investigated, in addition to the effectiveness of probiotic and prebiotic products, the effects of probiotic and prebiotic strains over microbiota, and their impact on the health status of the participants. The 15 RCTs were performed in populations with different gastrointestinal conditions, 7 in IBS [78,79,80,81,82,83,84], 2 in celiac disease [85,86], 2 in constipation [87,88], 1 in GFDs without constipation [89], 1 in lactose intolerance [90], 1 in premature infants with low degree of maturation of the gut microbiota [91], and 1 in functional diarrhea [92].

It should be noted that, of these 15 aforementioned clinical trials, within the scope of the microbiota, only two used a symbiotic mixture, one with *Lactobacillus* + *Bifidobacterium* + frutooligosaccharides [92], and the other one with prebiotic substrates + *Bifidobacterium* + *Lacticaseibacillus* + *Lactobacillus* + *Ligilactobacillus* [87]. This limited the results with regards to the use of prebiotics, and thus it is not possible to evaluate their interactions with the gut microbiota.

Table 3 describes the results obtained in the scope of this study, considering the changes in microbiota with the use mainly of probiotics through proposed mechanisms such as maintenance of microbiota stability, increase in beneficial bacteria, and decrease in pathogenic bacteria, by comparing the active group and placebo groups.

From the results obtained, it is possible to infer that the administration of probiotic strains confers some changes in the microbiota, through different mechanisms. Among these, the increase in beneficial bacteria was seen in 10 RCTs (66.7%), as well as the decrease in pathogenic bacteria reported in 4 (26.7%) of them. This ratio “increase in beneficial bacteria/decrease in pathogenic bacteria” may allow the microbiota to be kept healthy, thus resulting in benefits for the patients.

The process of maintaining or increasing stability was also considered and was verified in 7 (46.7%) of the RCTs analyzed, as the intention of the administration of the test products is that they can trigger beneficial changes in the microbiota without compromising its stability and diversity, which was confirmed in these studies. Regarding statistical significance, the differences established between the active group and the placebo group were statistically significant (*p* < 0.05) in 12 (80.0%) of the RCTs, thus confirming the hypothesis that supplementation with the products analyzed was related to the gut microbiota in most of the treated studies.

In the RCTs under evaluation, despite the existence of limitations such as the complexities in the analysis of microbiota attributable to the existence of several species, it is admitted that the symptomatic improvement associated with the disorders under study may be related, in part, to the ability of probiotics to modify intestinal microbiota.

However, further studies are necessary to obtain more representative results aiming at determining which are the most specific changes triggered by different probiotic species and defining for a given disease the correct species to be administered. Similar studies in the context of prebiotic species used in isolation or in association are necessary to understand their role in gut microbiota and are presently lacking.

### 3.2. Variables Associated with the Therapeutic Effectiveness and Long-Term Effects of Probiotics and Prebiotics

Among the 49 RCTs selected, 40 were analyzed in relation to the following data: age, gender, clinical status, number of individuals in the sample, duration of the study, pharmaceutical form, dose administered of the probiotic and/or prebiotic species, results obtained, statistical significance, and study limitations. Among the entire set of studies, 21 concerned IBS [78,89,93,94,95,96,97,98,99,100,101,102,103,104,105,106,107,108,109,110,111], 9 IBS-D [82,84,112,113,114,115,116,117,118], 1 IBS-M [115], 4 IBS-O [80,81,119,120], 3 constipation [88,121,122], 1 celiac disease [85], 1 diverse gastrointestinal symptoms [123], and 1 constipation specifically in children [87].

Table 4 describes the results obtained from the analysis of the RCTs analyzed, considering the samples number, administered products, treatment duration, symptomatic results, and statistical significance of the findings.

The selected studies that did not specifically concern IBS were included in the analysis because of their similar symptomatology. Among these studies, there were three placebo-controlled clinical trials analyzing chronic constipation, with *Bifidobacterium animalis* being administered in one of them [122], *Lactiplantbacillus plantarum* in the second [88], and in the third, a symbiotic mixture of *Bifidobacterium* + *Lactobacillus* + *Streptococcus* + fructooligosaccharides species [121], verifying the following as a result of supplementation: increased frequency of defecation, decreased feeling of incomplete evacuation, and some improvements in the Bristol Scale when comparing to placebo. In a clinical trial on celiac disease with specific symptoms of IBS, a mixture of *Lactobacillus* + *Bifidobacterium* species was administered, resulting in a significant decrease in the overall severity of symptoms and improvement in the Bristol Scale, having obtained a statistically significant *p*-value (*p* < 0.001), established by the Wilcoxon test through comparison between groups [85].

Concerning gastric symptoms such as postprandial discomfort, a significant relief (*p* < 0.05) of discomfort was noted after treatment with *Streptococcus termophilus*, compared to the placebo, determined through Wilcoxon and chi-square tests [123].

Finally, in a clinical trial carried out in children with constipation who received a symbiotic mixture of prebiotic substrates and several probiotic strains of *Bifidobacterium* + *Lacticaseibacillus* + *Lactobacillus* + *Ligilactobacillus,* a significant association (*p* < 0.05), determined by the Clopper–Pearson method, was observed between the treatment with an increased weekly frequency of spontaneous bowel movements [87].

The aforementioned findings suggest that by evaluating the effects of probiotics and prebiotics over gastrointestinal conditions, it may be possible to infer what GFDs-related symptoms can be targets to consider in the development of future research projects and guidelines. Studies carried out in children are also important because the therapeutic options for this population are often limited.

From the analysis of the obtained data among the total RCTs, it was verified that prebiotics were used in only six studies, of which four were in IBS, one in chronic constipation in adults and one in constipation in children, in association with probiotic strains. In one of these trials, the combination of the prebiotic inulin + *Lactobacillus* + *Bifidobacterium* resulted in significant decrease of flatulence when compared to the placebo [94]. In another trial [119], a comparison was made between the individual administration of probiotic versus the association of the same probiotic with polydextrose; it was observed in both samples, a significant reduction of fecal pH and gastrointestinal transit time, increased frequency of complete evacuation sensation, and adequate stool consistency. Further studies are needed to investigate the role of prebiotics, in isolation or in synergy with probiotics, since prebiotics have been scarcely investigated as a treatment option for IBS, according to our findings.

In this study, the administration of individual probiotics or associations between different strains, constitute most of the analyzed RCTs (85%), predominating the administration of the genera *Lactobacillus* and *Bifidobacterium*.

From the analysis of our data, it was possible to infer that the administration of probiotic strains resulted in the improvement of several symptoms associated with IBS, such as abdominal pain, distension and noise, flatulence, alterations in intestinal transit, and stool consistency and shape, according to the evaluation by the Bristol scale. These data suggest that probiotics play a significant role in reducing and improving IBS symptoms, with statistically significant differences (*p* < 0.05) being observable in 36 (90%) of the RCTs. Although a representative percentage of trials demonstrated effectiveness of probiotics, unfavorable effects were also observed in the probiotic group in comparison with placebo [96], these negative results should not be discarded, since it is crucial to investigate which strains can give rise to detrimental effects.

The studies carried out in IBS-D [82,84,112,118] and IBS-O [81,93,119,120] raise interest for the different responses yielded by varied strains in different IBS subtypes. For instance, in the trial in which the effectiveness of *Escherichia coli* and *Enterococcus* in IBS was evaluated [103], no significant improvement was found in the overall IBS symptoms; however, through a more accurate evaluation, a significant decrease in abdominal pain was observed in IBS-D when compared to placebo (*p* < 0.001), determined by Mantel–Haenszel and chi-square tests. Taking these results into account, it is pertinent to carry out further studies on the different subtypes of IBS aiming to investigate not only species-specific behaviors, but also their effects over distinct IBS clinical patterns.

Emphasizing the importance of the gut–brain axis in GFDs, namely IBS, from the analysis of the selected RCTs, it was observed that in 10% of the studies [82,105,116,118], improvement in anxiety, stress, and depression levels were evidenced, as well as a significant increase in serotonin serum concentrations in the active group, having obtained a *p*-value of less than 0.005 by carrying out independent t-tests and Mann–Whitney U-tests in the study [105]. These results raise interest for the development of further studies in this area, considering that many of the GFDs are related with disorders in the gut–brain axis.

Invaluable data were also obtained in the analysis of RCTs in which the effects of probiotics and a diet with low FODMAPS showed reduction in the overall symptomatic severity with both treatments, suggesting the possibility of a complementary therapeutic approach against IBS-associated symptoms [109,115].

In one RCT involving co-administration of *Bifidobacterium* + *Lactobacillus*, a relevant symptomatic improvement in the active group occurred only from the 8th week on, although in a shorter span of four weeks flatulence relief was already observed in comparison with the placebo, triggering interest for a possible faster onset of action of probiotics in certain symptoms, which should be investigated [117].

However, as may be expected, therapeutic responses seem to vary among different trials, concerning the duration of supplementation and long-term effects. While in trial [80] probiotics induced a greater reduction of symptoms until 60 days and a maintenance of results at 30 days after discontinuation, in trial [99], overall severity of symptoms appeared in the first week of treatment, but at the end of the trial no differences between the active group and placebo were identifiable. Also, in trial [81] there was a greater increase in feces of the species administered to the active group; however, after treatment interruption, this effect vanished, evidencing only a short-lasting effect of the products. Thus, a fundamental research question for future projects should be the continuity of the beneficial effects in the follow-up period after treatment discontinuation. Of the evaluated RCTs, there was variation concerning the time span of product administration, with 7.5% of investigation groups choosing a period of less than 1 month, and only 2.4% more than 6 months. Most (80.5%) studies lasted between 1 and 3 months, and 10% for a period of 3 to 6 months. Unfortunately, among all the analyzed studies, only 10 (25%) guaranteed a follow-up period after the discontinuation of supplementation.

Table 5 describes the results obtained during the follow-up period after discontinuation of supplementation.

According to Table 5, in 10 RCTs there were discrepant results concerning follow-up after treatment. In 6 RCTs (60%) in the follow-up period there was maintenance of the positive effects conferred by the intervention, and in 4 trials (40%), after discontinuation, the differences between the active group and placebo were no longer significant.

Despite the efficacy demonstrated in improving various IBS symptoms in the references under study, the main limitations observed were short time spans of treatments and, in the rare studies that investigated the remaining therapeutic effects, the short follow-up period. Given the results obtained in the follow-up period and the chronicity that characterizes most gastrointestinal pathologies, including IBS, it would be pertinent that new trials had longer durations of treatments and follow-up after the product discontinuation, aiming to confirm their efficacy and safety for longer periods.

### 3.3. The Definition of the Ideal Dosage Has a Significant Relationship with the Effectiveness of Probiotics and Prebiotics

The colony-forming unit (CFU) is defined as the unit of measurement used to determine the number of viable bacteria capable of multiplying under controlled conditions, and this was the unit selected to define the doses of probiotics and prebiotics used in the RCTs analyzed [124].

Most RCTs used doses already pre-established by other studies carried out, or even from products already on the market. Table 4 shows that the range of doses used was between 10^7^ and 10^11^ CFU, with 50% of RCTs applying the dose 10^9^ CFU.

Three of the analyzed RCTs used a dose-response approach, comparing the results obtained between 10^9^ CFUs and 10^10^ CFUs. In two of these studies, there was symptomatic improvement without significant differences concerning the varying dosages used [108,118], however, in one of them, there was a more significant reduction in symptoms at the higher dose [82]. In this way, it would be pertinent to invest in more dose-response studies to confirm whether different doses can be necessary for attaining the therapeutic response, obviously controlling for the occurrence of adverse reactions.

Considering the results described in terms of improving IBS symptoms with the dosages used, we may infer their efficacy. However, one of the main limitations of the selected studies was that they were performed in isolated research centers, making it inappropriate to generalize their findings concerning dosage adequacy for the entire population, given the interindividual variability. Therefore, further studies, including phase 3 multicenter RCTs are still lacking.

### 3.4. Regulation and Quality Control of Probiotics and Prebiotics Are Essential to Ensure Their Effectiveness and Safety and to Understand Existing and Future Clinical Data

From the analysis of the selected RCTs in the present study, it became evident that a huge diversity of probiotics with different formulations were employed. However, there was scarcely any comparison of efficacy between different types of formulation. One study alone compared the efficacy of the probiotic *Bifidobacterium longum* and the postbiotic of the same species with heat-treatment, and the results showed beneficial effects in comparison with the placebo, but no significant difference between the two distinct active groups [116].

A review study [125] performed to identify the best selection of probiotic products, proposed that the formulation quality should be a crucial criterion, and their choice should consider the shelf life, the larger concentrations for longer periods of freeze-dried capsules in comparison with dairy products, the longer half-lives of enteric coated capsules versus non-enteric capsules, and that refrigeration in necessary for non-lyophilized capsules. Another study refers to the criteria for checking up quality assurance of probiotic products, highlighting acid stability and adhesion properties to intestinal mucosa, noticing that usually, the control of these products only depends on tests to ensure their viability, which should not be the only criterion to be considered [126].

A study on five probiotic products available in India and Pakistan claiming to contain *Bacillus clausii*, found that 80% of the analyzed preparations presented lower concentrations of the species than the listed information, and the presence of contamination by other non-*Bacillus clausii* bacteria [127]. Another study analyzed five over-the-counter products in the United States and confirmed that the tested CFUs corresponded to the label [128]. The discrepancy in these results assumes different levels of observation to regulatory criteria and quality control in diverse countries concerning the marketed probiotic and prebiotic products.

A review of existing online information on prebiotics and probiotics concluded that descriptions of the benefits of these products outweigh descriptions of risks, particularly regarding the diffusion of presently available information related to adverse reactions [129]. Although most studies suggest that the level risk with the use of probiotics and prebiotics for healthy individuals is low, some adverse reactions have already been described (Table 1), mainly in immunocompromised individuals. From the analysis of the selected RCTs, only 10 (25%) studies reported adverse events, mostly gastrointestinal symptoms such as bloating, abdominal discomfort, and gastrointestinal alterations. Although these symptoms are considered low risk, the general population must be informed of putative risks, especially for immunocompromised individuals.

Currently, the regulatory criteria and quality control of probiotics and prebiotics are not well established, differing from country to country, and it would be wise to check out the existing information about these products to choose the more appropriate products for the entire population. Well-informed health professionals are expected to be the agents of clarification on this topic, helping select between these products in terms of benefits and risks.

## 4. Future Perspectives

This work can be seen as a starting point to trigger interest in the development of more multicenter RCTs that investigate the interactions between the various probiotic and prebiotic species and the gut microbiota, with adequate intervention and follow-up periods and the elaboration of more dose-response assays in larger samples and in more heterogeneous populations to evaluate the interindividual variability. It is imperative to reach a global consensus to guide health professionals, and the development of new studies may result in more cohesive guidelines that specify the therapeutic indications of probiotics and prebiotics.

## 5. Conclusions

In conclusion, the findings obtained in this study confirm the effectiveness of probiotics in IBS, even though there is still the need to refine knowledge about the interaction between probiotic strains and intestinal microbiota, the long-term effect of these products, and the definition of the most appropriately individualized dosages.

Probiotics and prebiotics can be seen as useful, either individually or as a complementary treatment. Additional multicenter RCTs with well-defined protocols, longer durations of treatment and follow-up, for each probiotic species, with different dosages for specific IBS presentations, and considering inter-individual variability would bring precious information. Considering the current diversity of products containing probiotics and/or prebiotics in food supplements, it is essential that the action of regulatory entities guarantees the quality and safety of these products on the market, provides a wide investigation of the risks of adverse reactions, and presents the parameters to be taken into account in their quality control. In addition to quality and safe control, regulators and health professionals should play an important role in the transmission of information.

## Figures and Tables

**Table 1:** Adverse effects described with the use of probiotic species.

Probiotic	Population	Condition for the Administration	Reported Adverse Effect	References
*Saccharomyces* *boullardi*	Immunocompromised; Neutropenia; Central venous cathether fungal infection	Treatment or prevention of diarrhea	Fungemia	[24,25,26,27]
*Saccharomyces* *cerevisae*	Newborns			[28]
*Lactobaccillus* *rhamnosus*	11-month-old baby with short bowel syndrome	Diarrhea resulting from tube feeding	Bacteremia	[29]
	Woman undergoing aortic valve replacement	Perioperative antimicrobial prophylaxis supplementation	Sepsis	[30]
	Man with mitral valve regurgitation	Preserving the intestinal flora after antibiotic treatment	Endocarditis	[31]
*Lactobacillus* *acidophilis*	5-year-old child	Complement in the treatment of short bowel syndrome	D-lactic Acidosis	[32]
*Lactobacillus* *casei*	Immunocompetent patient	Complement in the treatment of diverticulitis	Bacteremia	[33]
*Bacillus* spp.	Cancer patients	Feeding tube-associated diarrhea	Bacteremia	[34]
*Bacillus subtillis*	Man with chronic lymphocytic leukemia	Treatment/Prevention of gastrointestinal disorders	Sepsis	[35]
Probiotic mixture of *Lactobacillus* + *Bifidobacterium* + *Streptococcus*	Patients with pancreatitis	Evaluation of the reduction of pancreatitis complications	Intestinal Ischemia	[36]
	From infancy to adolescence	Treatment of antibiotic- associated diarrhea	Gastrointestinal side effects	[37]
	Effects on cytokine secretion and dendritic cell function culminating in immune system stimulation			[38]
*Lactobacillus reuteri*	Contains plasmid encoding chloramphenicol resistance which may result in genetic transfer of the probiotic for pathogenic bacteria			[39]

**Table 2:** Pharmacological measures for IBS.

	Abdominal Pain	SII-D	SII-O
1st line	Antispasmodics (mebeverine): promote the relaxation of the intestinal smooth muscle through drugs with anticholinergic and muscarinic properties [56,70]	Opioid antagonists (loperamide): Prolong intestinal transit [70]	Osmotic laxatives (macrogol): improves the frequency and consistency of stools and has fewer adverse effects than other osmotic laxatives [56,71]
2nd line	Tricyclic antidepressants or serotonin reuptake inhibitors: have analgesic properties and act on intestinal motility [71,72]	Bile acid sequestrants (Cholestyramine): bile acid triggers diarrhea by stimulating colonic secretions [71]	Guanylate cyclase agonists: promote intestinal transit with an effect on abdominal pain and bloating [71]
		5-HT3 antagonists (Ondansetron): decrease motility and secretion in the colon [69]	
		Rifaximin: can modulate gut flora and has a low risk for bacterial resistance [71]	

**Table 3:** Results obtained in the context of changes in microbiota described in the literature.

Ref.	Features of the Samples	Main Findings	Outcomes Assessment
[88]	181 subjects between 18 and 65 years old with chronic constipation	There was an increase in beneficial bacteria (*Lactiplantibacillus plantarum* and *Ruminococcus_B gnavus*) and a decrease in pathogenic bacteria (*Oscillospiraceae* sp., *Lachnospiraceae* sp. and *Herelleviridae*).	Assessed by NMDS analyses of fecal samples
[87]	64 children between 3 and 17 years old with constipation	There was an increase in beneficial specific bifidobacteria, and their maintenance over time, increasing the stability of the microbiota.	Measured by the analysis of fecal samples
[78]	86 subjects between 20 and 65 years old with IBS	There was an increase in stability with probiotic supplementation.	Assessed by a custom-made agilent microarray designed to cover the diversity of intestinal microbiota
[79]	55 subjects between 20 and 65 years old with IBS	Unexpectedly there was greater increase in *Bifidobacterium* spp. in the placebo group; however it may have been attributable to a competition between the administered species and others already detected at baseline in the placebo group, and with the supplementation a stability of the bacterial groups was observed.	Measured by real-time quantitative polymerase chain reaction
[80]	150 subjects between 18 and 65 years old with IBS-C	There was an increase in *Lactobacillus* spp. and *Bifidobacterium* spp. during treatment, stabilizing the gut microbiota.	Fecal microbiology analysis was assessed by quantitative PRC
[89]	68 subjects with a mean age of 37 years old with functional bowel disorders	An increase in *Bifidobacterium lactis* was observed.	Fecal samples were collected and analyzed using DNA-base methods
[81]	30 subjects between 18 and 65 years old with IBS-C	There was an increase in *Lactobacillus acidophilus* and *Bifidobacterium animalis.*	Fecal samples were collected and analyzed using real-time PCR
[84]	200 subjects between 18 and 65 years old with IBS-D	There was a reduction in *Clostridium sensu stricto* after treatment with *Clostridium butyricum.*	Stool samples were collected and analyzed using DNA-base methods
[82]	307 subjects between 18 and 70 years old with IBS-D	There was an enrichment of *Lactiplantibacillus* and *Lactobacillus plantarum* at the highest dose of the active group, as well as the maintenance of the stability and diversity of the microbiota.	Assessed by sample DNA isolation and quantification
[83]	42 subjects with a mean age of 46 years old with IBS	There was a decrease in *Ruminococcus torques.*	Measured by extraction and purification of DNA from fecal samples
[85]	109 subjects over 18 years old with celiac disease with IBS type-symptoms	There was an increase in *Lactobacillus*, *Lactococcus*, *Streptococcus*, *Staphylococcus* and *Bifidobacterium* in the active group.	Measured by DNA and RNA extractions from fecal samples
[91]	Extremely premature infants born at less than 1000 g birth weight and less than 29 weeks	There was an increase in the stability and interconnectivity of species supplemented in premature babies.	Assessed by strain-specific real-time PCR
[86]	40 subjects between 1 and 19 years old with celiac disease	There was an increase in Firmicutes, ensuring the stability of the microbiota with the maintenance of the Firmicutes/Bacteroidetes ratio.	Evaluated by DNA extraction from fecal samples
[90]	135 subjects between 20 and 67 years old with lactose intolerance and functional gastrointestinal symptoms	There was an increase in *Bifidobacterium* and a decrease in *Klebsiella*, *Serratia* and *Enterobacter* in the active group.	Evaluated by RNA extraction from fecal samples
[92]	39 subjects with mean age of 49.8 with functional diarrhea	There was an increase in *Lactobacillales* in the active group.	Assessed by DNA extraction from fecal samples

**Table 4:** Results obtained from the analysis of RCTs under study.

Ref.	Features of the Samples	Products Administered and Respective Doses	Treatment Duration	Results	Outcomes Assessment
[88]	163 subjects between 18 and 65 years old with chronic constipation	Powder in sachet of *Lactiplantbaccilus* (10^11^)	1 month	Greater increase frequency of defecation in the active group	All subjects completed an electronic stool diary during the study
[87]	64 children and adolescents between 3 and 17 years old with com constipation	Powder in sachet of *Bifidobacterium* + *Lactobacillus* + *Lacticaseibacillus* + *Ligilactobacillus* + prebiotic substrates (10^10^)	3 months	Increase in weekly frequency of spontaneous bowel movements greater in the active group	Parents reported in a logbook the daily frequency and consistency of their child’s stool
[121]	60 men over 18 years old with constipation	Capsule of *Bifidobacterium* + *Lactobacillus* + *Streptococcus* + frutooligosaccharides (10^8^)	1 month	Greater increase frequency of defecation and improvement in the Bristol scale in the active group	Assessed by “patient assessment of constipation symptoms questionnaire” and Bristol stool form scale
[122]	49 women between 10 and 50 years old with constipation	Solution of *Bifidobacterium* (10^7^)	2 months	Greater increase frequency of defecation and improvement in the Bristol scale in the active group	Assessed by Bristol Stool Form Scale
[123]	100 subjects between 20 and 64 years old with gastric symptoms	Solution of *Streptococcus* (10^7^)	1 month	Greater relief of postprandial discomfort in the active group	Evaluated by Gastrointestinal Symptom Rating Scale and Frequency Scale for Symptoms of Gastroesophageal Reflux
[85]	109 subjects over 18 years old with celiac disease	Powder in sachet of *Lactobacillus* + *Bifidobacterium * (10^9^)	2 months	Greater decrease in overall symptom severity and improvement in the Bristol Scale in the active group	Assessed by IBS Severity Symptom Score and Bristol Stool Form Scale
[93]	103 subjects over 18 years old with IBS	Capsule of *Lactobacillus* + *Propionibacterium* + *Bifidobacterium* (10^9^)	6 months	Greater reduction of abdominal pain, distension, rumbling, and flatulence in the active group	Abdominal symptoms were followed by a symptom diary
[94]	64 subjects between 18 and 75 years old with IBS	Powder in sachet of *Lactobacillus* + *Bifidobacterium* + prebiotic inulin (10^9^)	1 month	The reduction in the severity of flatulence was greater in the active group	Evaluated by a daily diary, Bristol Stool Form Scale, and Visual Analogue Scale
[95]	52 subjects between 18 and 75 years old with IBS	Medical device with *Bacillus coagulans* + simeticone	1 month	Greater reduction in bloating and abdominal discomfort in the active group	Evaluated by Visual Analogue Scale
[112]	400 subjects between 18 and 55 years old with IBS-D	Capsule of *Bacillus* + *Bifidobacterium* + *Lactobacillus* + *Lactococcus* + *Streptococcus* (10^10^)	4 months	Improvement in abdominal pain, gastrointestinal changes and higher quality of life in the active group	Evaluated by IBS Symptom Severity Score and IBS Quality of Life questionnaire
[119]	163 subjects over 18 years old with IBS-O	Solution of *Lactobacillus* vs. solution of *Lactobacillus* + polydextrose	7 days	Decreased fecal pH, intestinal transit time, frequency of sensation of incomplete evacuation, and hard stools	Evaluated by stool samples, red carmine capsule method, and Garrigues constipation questionnaires
[96]	16 subjects between 18 and 75 years old with IBS	Capsule of *Lactobacillus* (10^10^)	1 month	The number of weeks with symptom relief was greater in placebo group than in the active group	Evaluated by an IBS sum score and Bristol Stool Form Scale
[113]	36 subjects between 18 and 55 years old with IBS-D	Tablet of *Bacillus* (10^9^)	3 months	Reduction of symptoms such as bloating, vomiting, diarrhea, abdominal pain, and improvement in stool frequency and consistency greater in the active group	Measured by modified gastrointestinal discomfort questionnaire, Bristol Stool Form Scale, and Visual Analogue Scale
[97]	153 subjects between 18 and 60 years old with IBS	Capsule of *Bacillus* (10^10^)	2 months	Greater reduction of symptoms such as abdominal pain, bloating, sensation of incomplete evacuation and flatulence in the active group	Evaluated by IBS symptoms score
[114]	30 subjects between 18 and 75 years old with IBS-D	Capsule of *Bifidobacterium* + *Lactobacillus* (10^10^)	1 month	Normalization of intestinal permeability and improvement of stool consistency, abdominal pain, diarrhea, and psychological well-being greater in the active group	Evaluated by Visual Analogue Scale, IBS quality of life questionnaire, and “yes” or “no” questions
[78]	86 subjects between 20 and 65 years old with IBS	Solution of *Lactobacillus* + *Propionibacterium* + *Bifidobacterium * (10^7^)	5 months	Greater reduction in pain, distension, rumbling, and flatulence in the active group	Symptoms were followed by a diary
[120]	41 women between 20 and 69 years old with IBS-O	Solution of *Streptococcus* + *Lactobacillus* (10^9^)	1 month	Reduction of maximal abdominal distension and verification of greater colonic acceleration in the active group	Measured by expiratory breath samples and radio-opaque marker ingestion
[98]	56 subjects over 18 years old with IBS	Capsule of *Bifidobacterium* + *Lactobacillus* (10^10^)	2 months	Reduction in overall symptom severity in both groups, but more significant in the active group at week 8	Evaluated by a questionnaire to assess IBS symptoms
[99]	74 subjects between 18 and 70 years old with IBS	Solution of *Streptococcus* + *Lactobacillus* + *Bifidobacterium* (10^7^)	2 months	Overall symptom severity reduction was more visible in the active group at week 1 of treatment, but no difference existed between the active group versus controls at the end of the treatment	Assessed by a daily questionnaire, Bristol Stool Form Scale, and Quality of Life Questionnaire
[100]	122 subjects between 18 and 68 with IBS	Capsule of *Bifidobacterium* (10^9^)	1 month	Greater reduction of pain, abdominal distension, and urgency to defecate in the active group	Evaluated by 7-point Likert scale
[101]	152 subjects between 18 and 65 years old with IBS	Suspension of *Lactobacillus* + *Enterococcus* (10^9^)	3 months	Greater reduction in the overall symptom severity in the active group	Measured by IBS Severity Symptom Score
[80]	150 subjects between 18 and 65 years old with IBS-O	Capsule of *Lactobacillus* vs. Capsules of *Lactobacillus* + *Bifidobacterium* (10^9^)	2 months	Symptoms reduction and improvements in Bristol Scale up to 60 days and maintenance up to 30 days after higher dose in active groups	Assessed by a questionnaire of symptoms, health-related quality of life questionnaire, and Bristol Stool Form Scale
[102]	40 subjects between 18 and 65 years old with IBS	Powder in sachet of *Bacillus* (10^9^)	3 months	Greater reduction of abdominal pain, rumbling, nausea, vomiting, anxiety, and improvement of intestinal transit and stool consistency in the active group	Evaluated by Digestive Symptom Frequency questionnaire, IBS Symptom Severity Score, Bristol Stool Form Scale, and Quality of Life questionnaire
[115]	34 subjects over 18 years old with IBS-D or IBS-M	Powder in sachet of *Streptococcus* + *Lactobacillus* + *Bifidobacterium* (10^9^) vs. Low in FODMAPs diet	1 month	There was a reduction in the overall severity of symptoms and an improvement in the Bristol scale in both groups, but without significant difference between groups	Assessed by IBS Severity Symptom Score, Bristol Stool Form Scale, and Quality of Life questionnaire
[116]	200 subjects between 18 and 65 years old with IBS-D	Capsule of *Bifidobacterium* (10^9^)	3 months	There was a reduction in overall symptom severity and anxiety scores and an improvement in stool consistency and higher quality of life in the active group	Measured by IBS Severity Symptom Score, Bristol Stool Form Scale, Quality of Life questionnaire, Abdominal Pain Numeric Rating Scale, and State-Trait Anxiety Inventory Adults questionnaire
[89]	60 subjects between 18 and 65 years old with IBS	Tablet of *Bifidobacterium* + *Lactobacillus* (10^11^)	2 months	Greater reduction of abdominal bloating, in the active group	Assessed by a seven-point scale
[117]	80 subjects between 18 and 60 years old with IBS-D	Powder in sachet of *Bifidobacterium* + *Lactobacillus* + frutooligosaccharides (10^9^)	2 months	Greater overall improvement in symptoms in the active group at week 8 and faster relief of flatulence in the active group at week 4 were noticeable	Evaluated by IBS Severity Symptoms Score
[81]	30 subjects between 18 and 65 years old with IBS-O	Solution of *Streptococcus* + *Lactobacillus* + *Bifidobacterium* + dietary fiber (10^7^)	1 month	Increase in species in feces higher in active group; however, after discontinuation they returned to initial values	Participants collected their stool samples, and they were analyzed by real-time PCR
[103]	389 subjects over 18 years old with IBS	Probiotic lysate of *Escherichia coli* and *Enterococcus* (10^7^)	7 months	The improvement in the global assessment did not obtain significant differences, except concerning abdominal pain in IBS-D	Measured by IBS Global Assessment of Improvement Scale and 11-point numeric rating scale
[104]	80 subjects between 30 and 60 years old with IBS	Capsule of *Lactobacillus* (10^9^)	2 months	Pain, bloating, and flatulence improved in both groups, in great numbers in the active group but without statistical significance	IBS symptom score was assessed with Visual Analogue Scale
[84]	200 subjects between 18 and 65 years old with IBS-D	Capsule of *Clostridium butyricum* (10^7^)	1 month	Improvement in quality of life, severity of symptoms, bowel habits, and higher stool frequency in the active group	Assessed by IBS Severity Symptom Score and Quality of Life questionnaire
[105]	104 subjects between 18 and 65 years old with IBS	Solution of *Lactobacillus* (10^9^)	3 months	Only the probiotic group significantly increased serotonin serum levels	Assessed with Center of Epidemiology Studies Depression Revised questionnaire and hormonal analysis
[106]	50 subjects between 18 and 70 years old with IBS	Capsule of *Lactobacillus* (10^8^)	1 month	Reduction in symptoms severity in both groups, without significant difference. Some improvement in abdominal pain in the active group in IBS-D	Measured by IBS Severity Scoring System and Gastrointestinal Quality of Life Index
[107]	240 subjects between 18 and 70 years old with IBS	Capsule of *Lactobacillus* (10^9^)	1 month	Greater improvement in abdominal pain and distension, sensation of incomplete emptying and higher stool frequency in the active group	Measured by a Visual Analogue Scale and the daily number of stools were registered at each visit
[108]	340 subjects between 18 and 65 years old with IBS	Capsule of *Lactobacillus* (10^9^) vs. (10^10^)	3 months	Reduced sensation of major abdominal pain with both doses in the active groups	Evaluated by IBS Symptom Severity Score and IBS Quality of Life questionnaire
[82]	307 subjects between 18 and 70 years old with IBS-D	Capsule of *Lactobacillus* (10^9^) vs. (10^10^)	2 months	Decreased symptom severity at both doses, but greater response with higher dose	Assessed by IBS Symptom Severity Score
[109]	133 subjects between 18 and 74 years old with IBS	Capsule of *Lactobacillus* (10^9^)	2 months	Greater overall symptom reduction in the probiotic group and the low-FODMAPs diet group compared to the normal diet	Measured by IBS Symptom Severity Score, IBS Quality of Life questionnaire, and Hospital Anxiety and Depression Scale
[118]	84 subjects between 20 and 70 years old with IBS-D	Capsule of *Lactobacillus* + *Pediococcus* (10^9^) vs. (10^10^)	2 months	Improvement in quality of life and greater gut-specific anxiety in the active group in both doses	Measured by Quality-of-Life questionnaire and Visceral Sensitivity Index Scale
[110]	38 subjects over 18 years old with IBS	*Bifidobacterium* (10^10^)	2 months	Greater improvement in anxiety, depression, and decreased increased amygdala activation in the active group	Assessed by functional magnetic ressonance imaging and hospital anxiety and depression scale
[111]	103 subjects between 20 and 65 years old with IBS	Capsule of *Lactobacillus* + *Propionibacterium* + *Bifidobacterium* (10^9^)	6 months	Greater reduction of pain, rumbling, bloating, and flatulence in the active group	The participants completed a symptom diary

Abbreviations: IBS: irritable bowel syndrome; IBS-D: diarrhea predominant IBS; IBS-M: IBS alternating between constipation and diarrhea; IBS-O: constipation predominant IBS; low-in FODMAPS diet: diet low in oligosaccharides, disaccharides, monosaccharides, and non-fermentable polyols.

**Table 5:** Results obtained in the follow up period after treatment discontinuation.

Ref.	Follow-Up Duration	Findings During Follow-Up
[88]	2 weeks	Abnormal stool frequency continued to improve significantly in the active group after the discontinuation of supplementation.
[98]	2 weeks	After the follow-up period, significant differences were no longer detected in both groups in terms of improvement in the severity of symptoms, but the total number of days with pain was only reduced in the active group.
[81]	2 weeks	After discontinuation of supplementation, the values for the probiotic species found in feces decreased, suggesting that treatment effects were transient.
[78]	3 weeks	The improvements obtained continued to be observed in the follow-up period.
[112]	1 month	The improvements obtained continued to be observed in the follow-up period.
[101]	1 month	The response to probiotics remained after treatment discontinuation, although there was increase of the clinical response in the placebo group, resulting in disappearance of the previously observed significant differences.
[80]	1 month	There was a maintenance of the effects obtained during the treatment.
[108]	1 month	There was a maintenance of the effects obtained during the treatment.
[85]	2 months	After follow-up, no significant differences previously observed between groups remained.
[115]	1 year	The trial was carried out with 1 year of follow-up using a web application, and whenever there was symptomatic worsening, patients performed a cycle of treatments for 4 weeks, and improvements in symptomatology were observed among them.

## Data Availability

No new data were created or analyzed in this study.
